# Editorial: Epithelial plasticity and complexity in development, disease and regeneration

**DOI:** 10.3389/fcell.2022.1105402

**Published:** 2023-01-12

**Authors:** Joan Li, Minoru Takasato, Qihe Xu, Roel Bijkerk

**Affiliations:** ^1^ Medical School, Faculty of Medicine, University of Queensland, Brisbane, QLD, Australia; ^2^ Laboratory for Human Organogenesis, RIKEN Center for Biosystems Dynamics Research, Kobe, Japan; ^3^ Renal Sciences and Integrative Chinese Medicine Laboratory, Department of Inflammation Biology, School of Immunology and Microbial Sciences, Faculty of Life Sciences an Medicine, King’s College London, London, United Kingdom; ^4^ Department of Internal Medicine (Nephrology) & Einthoven Laboratory of Vascular and Regenerative Medicine, Leiden University Medical Center, Leiden, Netherlands

**Keywords:** epithelia, development, plasticity, disease, regeneration

Epithelial plasticity, the ability of epithelial cells to change phenotypes, is a fascinating phenomenon that has been extensively studied for decades. Most commonly epithelial plasticity refers to the conversion between epithelial and mesenchymal phenotype, known as epithelial-to-mesenchymal transition (EMT) and mesenchymal-to-epithelial transition (MET). Both EMT and MET are common features in embryonic development, tissue responses to damage, e.g., inflammation, repair, and cancer. Under the Research Topic “*Epithelial plasticity and complexity in development, disease and regeneration*” a collection of original research articles and research report have been gathered addressing the fascinating and complex states of epithelial plasticity, their distinctions and functions, in human and other model systems.

Epithelial to mesenchymal transition (EMT) has been classically defined as a developmental program that is instrumental in early embryo patterning for many organs, characterized by epithelial cells losing cell-to-cell adhesion, epithelial tight junctions, and desmosomes. Evolutionally, EMT processes enabled organisms to acquire more-complex structures by creating mesenchymal cells of mesoderm from primitive ectoderm. EMT is a fundamental process in physiologic repair and pathologic fibrosis of tissues and organs. More recently it has been recognized that EMT also plays a critical role in facilitating the creation of a pro-tumoral microenvironment, promoting tumorigenesis and metastasis. Reorganization of intercellular junctions, particularly tight junctions is a key event of the EMT process during tumor progression. In this issue Neyrinck-Leglantier et al. published their research work investigating how the tight junction protein Zonula occludens-1 (ZO-1) is involved in modulating tumor microenvironment. Using both *in vitro* and *in vivo* models they demonstrated that relocation of membrane-associated ZO-1 towards the cyto-nuclear compartments can modulate the secretion of pro-inflammatory chemokines hence promote immune cell recruitment to the tumor microenvironment. This work provides further support to earlier studies which reported that significant reduction of ZO-1 membrane expression and relocation of cyto-nuclear expression were closely associated with enhanced expression of the pro-inflammatory cytokine interleukine-8 (IL-8) in cancer.

In recent years increasing evidence has demonstrated the novel aspects of epithelial biology challenging the traditional classical definition of epithelium. Reports of sub-specialized epithelial, transit-epithelial and the conversion between epithelial sub-types in response to injury promote further investigation into the full complexity of epithelial plasticity and potential novel functions of conventional as well as non-conventional epithelial cells. The epicardium, a mesothelial cell layer enveloping the heart, is a slightly different type of epithelium. It has been recognized as a crucial contributor to heart development and repair. Increased epicardial activity is reported to be associated with an improved reparative response of the mammalian heart. Epicardial epithelial to mesenchymal transition (epiMT) initiates epicardial migration, critical for heart development. It is proposed that similar signaling pathways could trigger epiMT following injury and regulate epicardium-driven repair of the heart. Dronkers et al. reported the findings of novel regulators of epiMT. Their study shows that independent of the commonly accepted EMT inducer TGFβ, Activin A and ALK4 signaling modulate epicardial plasticity and epicardial invasion in cultured embryonic mouse hearts.

Renal proximal tubular epithelial cells have been regarded as classical epithelium. But in recent years animal studies suggest that proximal tubular cells are plastic epithelial cells because they can change phenotype to present a scattered proximal tubular cell pattern in response to injury, possibly involved in the regeneration processes after tubular injury. This was further supported by the observation of multiciliated proximal tubular cells with motile cilia in kidney biopsies of patients with various kidney diseases. Eymae et al. reported the existence of multiciliated cells in patient’s kidneys, closely associated with expression of tubular injury markers and evidence of interstitial fibrosis. Mammalian kidney epithelial cells have been documented as containing a single non-motile primary cilium; the presence of multiciliated cells indicates that proximal tubular cells are able to transdifferentiate and change phenotype. This could be an adaptive response to injury to maintain tubular function and contribute to the post-injury survival or repair, or a maladaptive repair subsequently resulting in kidney fibrosis. The exact function and mechanism of proximal tubular cells converting into multiciliated cells are yet to be fully explored.

The most significant physiological functions of epithelial cells are fluid and solute secretion and absorption *via* its many membrane channels. Two typical ion channels, cystic fibrosis transmembrane conductance regulator (CFTR) and the epithelial sodium channel (ENaC), are broadly expressed and responsible for Cl− secretion and Na+ absorption, across the epithelium. In recent years it is suggested that the interaction between epithelial ion channels may provide an efficient mechanism driving epithelial plasticity and self-remodeling. In this issue, Wuchu et al. reported a novel biphasic regulatory role of ENaC in tuning CFTR expression, through Ca^2+^-modulated cAMP production, in cultured human endometrial epithelial cells and human bronchial epithelial cells using shRNA-based stable knockdown of ENaC. Increased understanding of the regulatory mechanisms underlying the switch between secretion and absorption of epithelial cells will help to address the environmental dynamics which has both physiologic and pathophysiologic significance in function restoration and repair following injury.

In addition to phenotypic changes, epithelial cell proliferation and migration have always been regarded as critical for normal tissue homeostasis and for post-injury repair. Mechanisms that can trigger effective directional migration of endogenous epithelial cells are important in would healing process. The effect of physiologic electric fields (EF) on cell migration has been studied extensively in development and wound healing. Reports suggest that EF can direct renal epithelial cell migration directly contributing to the tubular regeneration process following acute kidney injury. The study published by Guan et al. suggests that in cultured HK-2 and HEK-293 human renal tubular epithelial cell lines, EFs can augment the rate of cell migration through activation of Erk1/2 and p38 mitogen-activated protein kinases and Akt signaling. Following this observation, exposure to EF also promotes wound healing by enhancing renal epithelial cell migration *in vitro*.

Taken together, though the micro-environmental signals and responding machinery enabling phenotypic and functional changes of the epithelial cells are yet to be fully understood, the significance of epithelial complexity and the importance of epithelial plasticity have been recognized and highlighted in the contexts of inflammatory response, cancer progression and wound repair.

